# The Poetic Life Narratives of Midlife and Older Bisexual Adults: Struggle, Survival, and Healing in Three Acts

**DOI:** 10.1093/geroni/igaf122.1071

**Published:** 2025-12-31

**Authors:** Sarah Jen, Olivia Lafountain, Xavier Noriega, Austin Oswald, Zhiqi Yi

**Affiliations:** University of Kansas, Lawrence, Kansas, United States; University of Kansas, Lawrence, Kansas, United States; University of Kansas, Lawrence, Kansas, United States; Dalhousie University, Halifax, Nova Scotia, Canada; University of Kansas, Lawrence, Kansas, United States

## Abstract

While empirical discourses surrounding bisexuality and aging often focus on challenges, stigmas, and disparities, analyses of bisexual coping and survival offer potential counternarratives of hope and possibility. The purpose of this analysis is to narrate stories of coping and survival among midlife and older bisexual individuals in the context of their life course experiences. Findings are presented in a series of three found poems, or poems created from the direct quotes of study participants, which are followed by interpretations of the quotes in the broader context of participants’ lives. Each poem depicts a particular “act” of life, breaking up the narratives into three distinct stages of meaning-making. Act I: The Struggle captures early- to midlife experiences fraught with a sense of confusion, lack of belonging, mental health challenges, and interpersonal conflict. In Act II: Coping and Survival Looks Like…participants reflect on the personal strengths, skills, and relationships that allowed them to move through their most significant challenges. Act III: Healing Bisexual Futures, presents reflections on what bisexuality means to the participants at this stage of their lives and how they envision and dream of their own futures. We see this streamlined narration of an often complex and messy life history as an agentive story-telling process in which bisexual individuals seek meaning, direction, and cohesion in the telling of their life experiences. Creative research methodologies are particularly well-suited to capturing the narrative and lyrical nuance in such stories, as well as their emotional poignance.

